# Intra-Cranial Complications of Ear Disease: A Discussion at the Bristol Medico-Chirurgical Society, March 13th, 1912

**Published:** 1912-06

**Authors:** J. Michell Clarke, J. Lacy Firth


The Bristol

Medico-Chirurgical Journal.

*"Scire est nescire, nisi id me
Scire alius sciret."*

JUNE, 1912.

### INTRA-CRANIAL COMPLICATIONS OF EAR DISEASE.

### A DISCUSSION AT THE BRISTOL MEDICO-CHIRURGICAL SOCIETY, MARCH 13TH, 1912.

INTRODUCED BY

J. MICHELL CLARKE, M.A., M.D., F.R.C.P.,

AND

J. LACY FIRTH, M.S., F.R.C.S.

DR. MICHELL CLARKE said: Without taking up time by preliminary observations, I proceed at once to deal with the most important intra-cranial complications of ear disease, which are, of course, meningitis, abscess and sinus phlebitis.

Certain symptoms of cerebral disturbance may occur in disease of the ear, especially of the internal ear, only, and the first step is to determine whether there is actually intra-cranial disease. Even in this preliminary step in diagnosis the decision may be by no means easy. Although the onset of disease of this kind is so often insidious, by the time we are called upon for such a decision the symptoms are generally grave and acute.

Some assistance may be had from the character of the otorrhœa, for a discharge which is septic is far more likely to lead to intra-cranial complications.

Very severe symptoms may be present in otorrhœa with mastoid disease alone. Thus Byrom Bramwell[1] gives severe headache, pains radiating over the side of the skull or within the head, vomiting, giddiness, ataxia, nystagmus, and even epileptiform convulsions and optic neuritis, as occurring thus. Such symptoms, with exception of giddiness, ataxia and nystagmus, would, however, point rather to intra-cranial abscess than to ear disease as the cause. The difficulty of distinction is increased because (1) the patient is too ill as a rule to carry out the rather elaborate tests which are used to distinguish the vertigo and nystagmus originating in internal ear disease from those of cerebellar origin, although persistent nystagmus on the whole indicates disease of the cerebellum, and (2) because optic neuritis may be present in chronic otorrhœa. Ballance[2] states that the optic neuritis in such cases is of slight intensity, may be present for months without increasing, and subsides on free removal of temporal bone disease. Its persistence, therefore, after a complete operation of this kind would suggest an intra-cranial origin for it. These cases of optic neuritis in ear disease without signs of intra-cranial complication are very important, and I hope that subsequent speakers in this discussion may be able to throw some light on their frequency and severity. Their chief importance lies in the fact that if unrelieved optic atrophy and blindness may result. There are further those cases of optic neuritis with mastoid disease and indefinite signs of some intra-cranial complication which lead to so much difficulty. In a recent case of Dr. Firth's (a girl of about eleven years), operated on for mastoid disease, there was optic neuritis, most intense on the ipsolateral side, the head was kept bent towards the same shoulder, and the face turned to the opposite side, together with

[1] Allbutt and Rolleston's *System of Medicine*, vol. viii., 1910, p. 250; "Abscess of the Brain."

[2] Allbutt and Rolleston's *System of Medicine*, vol. iv. part ii., 1908, p. 475.

intense headache, vertigo, slight nystagmus, especially to ipsolateral side, difficulty in standing, and tendency to fall and to stagger towards the same side. The cerebellum was explored without result, and the child recovered gradually.

It is also important to remember here that there may be suppuration in the internal ear and an intra-cranial complication, and yet the membrana tympani may be intact. I do not propose to deal with septic sinus-phlebitis, but I should like to ask those with more experience whether repeated rigors and a high temperature of 104° and 105° occur in uncomplicated mastoid or internal ear suppuration, or whether such symptoms are diagnostic of septic sinus thrombosis in the absence of tenderness and swelling over the jugular vein?

In this stage of doubt, or of suspended decision as to the presence of an intra-cranial complication, Sir V. Horsley[1] has rightly insisted upon the necessity for a thorough neurological examination to be carried out and repeated from day to day, including daily observation of the optic discs until a diagnosis is arrived at.

Having decided that an intra-cranial complication exists, the next point to settle is whether it is due to the ear disease present. This is not always easy. A patient with chronic otorrhœa may get an attack of influenza or be suffering from uræmia; in both cases cerebral symptoms may occur which may closely simulate meningitis, or the latter may be actually present as a consequence of influenza. Further, a child with chronic otorrhœa may develope polio-encephalitis. This affection is especially apt to follow the specific fevers, for instance, measles. Measles not infrequently gives rise to middle ear disease and otorrhœa, so that both affections might own the same cause; but in such a case help would be derived from the knowledge that intra-cranial abscess, at any rate, practically never occurs as a result of recent otorrhœa, but is a consequence of otorrhœa of some standing. Polio-encephalitis, as Batten has shown, may attack the cerebellum, when the symptoms will be those belonging to this organ. Polio-encephalitis is, however, a rare

[1] *Proc. Roy. Soc. Med.*, 1911–12, v. (Otolog. Sect.), 45.

disease, and as a complication with otorrhœa would be very seldom. More frequent and important is the combination of otorrhœa with tuberculous meningitis; the otorrhœa may or may not be due to tuberculous ear disease. In my experience it is not at all uncommon for patients with tuberculous meningitis to be suffering from otorrhœa, and this is one possible route for tuberculous infection of the meninges. The diagnosis can generally be made, and the independent origin of the meningitis from the otorrhœa determined; and this is most important, for it saves the patient from possible operation, which could only hasten the end. The chief points in the diagnosis are the history of prodromal symptoms as compared with the acute onset of septic meningitis; the seat of the meningitis is generally at the base, and the characters of the cerebro-spinal fluid obtained by lumbar puncture, showing a lymphocytosis and the presence of tubercle bacilli.

If we have come to the conclusion that an intra-cranial complication directly due to the ear disease is present, we have then to determine its nature. Omitting sinus thrombosis, the distinction has to be made between abscess and meningitis. Sometimes this diagnosis is quite easy, more often it is extremely difficult, and there is the further complication that both abscess and meningitis may be present. The signs that would make the diagnosis of abscess easy are a subnormal or normal temperature, a slow and full pulse, slow but more or less normal cerebration, optic neuritis more marked on one side, headache, vomiting, giddiness, apathy, a foul tongue, progressive emaciation, with or without the special signs which enable the abscess to be localised in one of its two common situations—the cerebellum or temporo-sphenoidal lobe. In the diagnosis of meningitis most important aids are the temperature, the pulse, and the examination of the cerebro-spinal fluid. The temperature in meningitis is raised, in abscess normal or subnormal. This is a point, however, which I hope subsequent speakers will deal with, especially as to the occurrence of a raised temperature in intra-cranial abscess, more especially in its early stages. It is certain that reliance on the temperature alone may lead to error. Thus Dr. Byrom Bramwell[1] quotes a case in which there were three acute abscesses in the brain with suppurative meningitis at the base and great increase of the intra-cranial pressure. The temperature throughout the whole course of the case was subnormal, and the pulse never over 84. Dr. Logan Turner[2] referred to a case which suggested meningitis in the posterior fossa, but in which a cerebellar abscess was really present. The cerebro-spinal fluid was turbid, contained albumin and a large number of polymorphonuclear cells. The temperature was high, the pulse rapid, together with rigidity of the neck, double Kernig's sign, and a leucocytosis of 22,000. *Post-mortem*, a large cerebellar abscess was found lying close to the surface of the lobe and leaking into the meningeal spaces through a small aperture. No meningitis could be detected macroscopically. The greatest aid is obtained from the results of lumbar puncture. In meningitis the cerebro-spinal fluid is more or less turbid, contains albumin, and an increased number, often greatly so, of leucocytes, whilst the causative organism may be recovered from it, and the cause thus fully cleared up. In abscess, on the other hand, although there are exceptions, as in the case related by Dr. Logan Turner,[3] the cerebro-spinal fluid is generally clear, with no, or only a very slight, increase in the number of cells. Again, in meningitis the pulse is rapid and often irregular. Optic neuritis, if present, is bilateral, and other nervous signs, such as any alteration in reflexes that may be present, or in the size of the pupils, are bilateral. Kernig's sign I have not found of very great value as a distinctive sign.

A few words may be said as to the condition known as serous meningitis. This is an effusion into the meningeal or ventricular spaces excited by the presence of a pyogenic focus in their vicinity. According to Dr. Ballance,[4] it may be general, but sometimes the serous effusion is locally limited, and this may occur over the cerebellum and temporo-sphenoidal lobe, and give rise to difficulty in the diagnosis from abscess. He says

[1] *Loc. cit.* [2] *Proc. Roy. Soc. Med.*, 1911–12, v. (Otolog. Sect.), p. 62.
[3] *Loc. cit.*, p. 63. [4] *Loc. cit.*

that serous meningitis occurs chiefly in young adults, and that its signs are headache, drowsiness, a rise of temperature with a relatively slow pulse, restlessness, vomiting, and a dilated and sluggish pupil. Relief is given by lumbar puncture, the cerebro-spinal fluid being under considerable pressure, and containing no excess of or few leucocytes. Lumbar puncture is therefore of great value in diagnosis.

I do not think it necessary to do more than allude to the combination of posterior basic meningitis with otorrhœa, as the symptoms are distinctive, and lumbar puncture establishes the diagnosis.

The symptoms of intra-cranial abscess will differ according to its character, whether it is acute or chronic, and whether it is encapsuled or not. A chronic encapsuled abscess may give rise to no symptoms. There are two general symptoms on which I hope subsequent speakers may give information. First, as to the presence of fever in abscess. I gather from the recent discussion at the Otological Section of the Royal Society of Medicine that fever is absent, except possibly in the initial stages of abscess. Secondly, as to the value in diagnosis of vomiting, I have seen it stated that in cases where an abscess is extending vomiting is severe and persistent. I am not sure from my own experience that this is the case. At any rate, in two or three cases the vomiting, which had been constant and severe before, ceased shortly after admission to hospital, although the other symptoms were unchanged, and I am inclined to think that the change to the orderly *régime* of a hospital and careful regulation of diet are sufficient by themselves to check vomiting in some of these cases.

Intra-cranial abscess as a complication of ear disease is almost invariably, if single, situated either in the cerebellum or temporo-sphenoidal lobe, and on the same side as the ear disease. It is worth bearing in mind that with disease in the external ear or mastoid the abscess is generally in the cerebellum, and with disease in the tympanum in the temporo-sphenoidal lobe. The diagnosis between abscess in these two situations may be fairly easy or so difficult that, although we are able to say that an intra-cranial abscess is present, it is almost impossible to be certain as to its position.

In either the cerebellum or the temporo-sphenoidal lobe an abscess may be entirely latent. In the cerebellum it will not give rise to characteristic signs if small and so situated as not to press upon the intra-cerebellar tracts of the superior and inferior cerebellar peduncles or of the nucleus dentatus. In the temporo-sphenoidal lobe there is a large "silent area," and unless the abscess is large, so as to cause pressure upon the Rolandic cortex or internal capsule, or is situated in the tip of the lobe, which is concerned with taste and smell, or on the left side in the posterior two-thirds of the superior temporal convolution, the auditory centre, it will give rise to no localising symptoms.

With regard to abscess in the lateral lobe of the cerebellum, in addition to the general signs of intra-cranial abscess, the localising signs, more or fewer of which may be present, are similar to those which we look for in a tumour in the same situation, but are rarely so well shown as in tumour, and, on account of the patient being more acutely ill in abscess from ear disease, cannot as a rule be so thoroughly investigated. Very often we have to be content with what we can find out from those symptoms which can be investigated with the patient lying in bed, and we are therefore deprived of the assistance to be obtained in diagnosis of cerebellar disease from the characteristic disorders of equilibration, attitude, position of the head, and gait. The most important signs are vertigo, ipsolateral paresis of the limbs [that is to say, if the ear disease is unilateral, on the same side as the ear disease], ataxia in the ipsolateral limbs, some ataxy of movement, and the position of the patient, who lies curled up in bed on the normal side, and returns to this position when disturbed. With regard to the eyes, there may be optic neuritis, weakening of the conjugate movement to the ipsolateral side, less commonly they deviate to the opposite side, lateral nystagmus, with slower jerks of greater amplitude to ipsolateral side. The pupils are unaffected, unless both are dilated from general rise of intra-cranial pressure. There is sometimes paralysis of the VIth nerve. The knee-jerks vary, sometimes the jerk is increased on the ipsolateral side ; the superficial reflexes are unaltered. The general signs of an intra-cranial abscess have been mentioned ; but the position of headache in cerebellar abscess should be borne in mind, it is very often felt in the opposite frontal region. As to optic neuritis, as compared with abscess in the temporo-sphenoidal lobe, it comes on earlier and is more intense, and according to Sir Victor Horsley in either case it is more marked on the side of the abscess. The value of paralysis of the VIth nerve as a localising sign is much diminished by the facts that it is the first nerve to suffer in any cerebral affection which raises the intra-cranial pressure, that basic meningitis must be excluded, and that it is occasionally paralysed in ear disease without any intra-cranial complication, as in the case of a boy under Mr. Firth's care whom I saw recently. The position of the head, said to be held down towards the ipsolateral shoulder, with turning of the face towards the opposite side, which is given as a sign of lesion of a lateral lobe, I do not think is a reliable sign. Its value is especially doubtful in acute ear cases with mastoid operation, for then the head is sometimes kept in this position when there is no intra-cranial complication. In those cases where a patient is very ill, and just able to stand out of bed with support, the side towards which he tends to fall is not of much aid in diagnosis.

The paresis of the ipsolateral arm and leg with absence of facial paresis, a normal or increased knee-jerk, flexor plantar reflex, and normal abdominal reflexes is of great importance in the differential diagnosis from abscess of the temporo-sphenoidal lobe, for paresis due to the latter will be contralateral, and will affect the face, the contralateral abdominal reflexes will be absent, the plantar reflex extensor and the knee-jerk increased. Unfortunately the value of this distinction is rendered uncertain in double otorrhœa by the difficulty of determining which ear is responsible for the intra-cranial lesion ; but where one ear alone is affected the homolateral paresis of cerebellar disease contrasted with the contralateral of a temporo-sphenoidal abscess, together with the other accompanying and distinctive signs of these two lesions respectively, is of great value.

A further caution must be given with regard to the characters of a hemiplegia, that should the cerebellar abscess be chronic and have attained a very large size, it may press upon the underlying pyramidal tract in the medulla, and therefore give rise to a paresis of the arm and leg, with an extensor plantar reflex and loss of abdominal reflexes on the opposite side. In this case some of the cranial nerves would be also involved, but the frequent affection of the VIIth in the Fallopian canal and of the VIIIth from the ear disease itself lessens the value in diagnosis of cranial nerve paralysis.

Sometimes most of the above signs of affection of the lateral lobe of the cerebellum are present in abscess, more often only a few occur. In a case that I saw with Mr. Morton some years ago the signs were unusually complete. The patient, a girl of 13, in addition to vomiting, persistent subnormal temperature, 97° F., pulse of 84, frequent fits, which suggested the cerebellar type of fit, emaciation, slow cerebration, deafness and discharge from the left ear, lay curled up on her right side in bed, with the right hand under her head, and constantly returned to this position when disturbed. There were also paresis and ataxy of the left arm, increased left knee-jerk, slight conjugate deviation of eyes to right, nystagmus, with more ample excursions to left, and right frontal headache. Double optic neuritis was present, and it is interesting to note that throughout in this case it was most intense in the right contralateral eye. Mr. Morton evacuated a large abscess in the left lateral lobe of cerebellum, and complete recovery ensued.

With regard to abscess in the temporo-sphenoidal lobe, it may be latent, because it lies in the "silent area," and this area comprises the greater part of the lobe, or because there is a free communication between the abscess cavity and the tympanum through which it can discharge. Unless the discharge is for some reason checked, there may then be no symptoms. The prompt appearance of cerebral symtoms when a communication of this kind is blocked is probably responsible for the old idea that it is dangerous to cure an otorrhœa. A distinctive sign of temporo-sphenoidal abscess is aphasia, in the form of word-deafness, and one symptom is said to be especially common, that is inability to name objects. From pressure upon the neighbouring angular gyrus in these cases there may be also word-blindness. If a true aphasia (word-deafness) can be distinctly made out, it is pathognomonic, and excludes the cerebellum as a possible seat of the abscess. It is, however, a symptom which is only available when the disease is on the left side. Further, when the patient is very ill, suffering from toxæmia or from a general increase of the intra-cranial pressure, there may be a state of mental confusion which may be mistaken for aphasia, and as the auditory centre only occupies the posterior two-thirds of the superior temporal convolution, it may easily escape damage even when the disease is on the left side. For the same reason that the centre for taste and smell is confined to the tip of the lobe, affection of these senses is not common.

The distinction between the hemiparesis or hemiplegia produced by a temporo-sphenoidal and a cerebellar lesion respectively has been already alluded to. It remains to be said that this symptom in abscess of the temporo-sphenoidal lobe is produced by involvement of the adjacent Rolandic area of the cortex or by pressure upon the internal capsule. In the former case the onset of the hemiplegia may have a definite march, affecting in order the opposite side of the face, the arm, and the leg. If, however, the hemiplegia is established by the time the patient comes under observation the history is rarely definite enough to determine this point. Sir V. Horsley, in the discussion at the Otological Section, also pointed out the importance of a careful investigation into those forms of sensation represented in the cortex, especially the distinction between two points as such and sense of localisation. He also pointed out that in cases of multiple abscess the second one is often in the parietal lobe, and that there may then be a rise of temperature on the opposite side of the body.

Lastly, from pressure upon the IIIrd nerve, the pupil on the side of a temporal abscess may be dilated and stabile, a valuable sign.

The above is a very brief summary of the signs which are of localising value in abscess in these two situations.

As has been already stated, in some of the symptoms the presence of double otorrhœa makes it difficult to determine their significance. It would be a great gain if the aural surgeon in double otorrhœa could give us certain indications as to which ear is the source of intra-cranial abscess; but unfortunately it seems that the ear which is not presenting the most active mischief at the time of observation may be the offender in this particular respect.

The diagnosis of abscess may be still further complicated by (1) the concomitant presence of meningitis, in which case the distinction could only be made by there being present definite localising signs of an abscess in addition to the general signs of meningitis, and (2) by the occurrence of encephalitis. I do not propose to take up more of your time by discussing the occurrence of abscess in more unusual situations, and will end this introduction to the discussion by emphasising, when an intra-cranial complication of ear disease is suspected, the value of an examination of the cerebro-spinal fluid, except in a case where an abscess in the cerebellum is fairly certain, in which case it is safer not to do a lumbar puncture, and of the risk of delay in operating when the diagnosis of an intra-cranial abscess has been arrived at. I should like to ask when a cerebellar abscess secondary to ear disease is present what is the best route of approaching it, whether, if there has been a mastoid operation, from the original opening or from a separate trephine opening in the occipital region. Lastly, if there is optic neuritis in ear disease without evidence of abscess or meningitis, and it persists after a free mastoid operation has been done, a decompression operation should be carried out.

Mr. Lacy Firth said: (1) **Inflammation of the outer surface of the dura mater and extradural abscess.**—In this complication of suppurative ear-disease the collection of inflammatory material, granulation tissue and pus, is epidural, lying between the surface of the temporal bone and the dura normally in contact with that bone. The incidence of the complication is almost doubly as frequent in males as in females. It affects individuals of all ages about equally. The causative inflammation most often involves the temporal bone itself, which is carious, necrosed, or ulcerated from cholesteatoma. More rarely the mucous membrane of the tympanum or antrum is alone involved.

It is an important point that pachymeningitis externa is more frequently met with as a complication of acute than of chronic otitis. The bone disease, when present, usually extends to the dura, but sometimes the path of infection is a fistulous tract passing from the ear cavities to the dura, perhaps arising from periphlebitis of vessels in the bone. Diplococcic infection is the most frequent. In some cases the extra-dural abscess may be secondary to sinus thrombosis, the inflammation passing from the sinus through its outer wall ; but often the reverse is the case, *i.e.* the extradural abscess is primary, and the thrombosis secondary. It is in the cerebellar fossa that the extension of inflammation is most frequently found, but it may be in the middle fossa, or in both. Sometimes very large collections of pus form in these situations. For example, the pus may extend up to the sagittal suture, or backwards to the torcular, or downwards to the jugular bulb and into the neck. Even retro-pharyngeal abscesses have been observed from this cause. The dura may withstand penetration of the inflammation or abscess to its inner surface for weeks, months or years if the pus can partially escape through the ear, but if not the further complications of sinus disease, brain abscess or meningitis will ensue.

Symptoms.—Frequently there are no symptoms of extra-dural suppuration over and above those of aural and mastoid inflammation. Fever is usually absent. In children fever is commoner than in adults, just as fever is commoner in them also from uncomplicated aural disease or from mastoid disease. Cerebral pressure symptoms are usually absent. There may, however, be headache, drowsiness, vomiting, slowing of the pulse, optic neuritis, stiffness of the neck, and even such disturbances as crossed paresis and speech troubles.

More important signs of extradural abscess, though seldom present, are swelling, pain and tenderness above the ear, due to penetration of the squamous portion of the temporal bone by the pus, or similar signs behind the ear, due to penetration of the abscess through the foramen of the mastoid emissary vein.

Diagnosis.—The cerebral complications mentioned above, viz. drowsiness, vomiting, optic neuritis and stiffness of the neck, do not enable a distinction to be made between this complication and those of brain abscess or meningitis. A very free outflow of pus from the ear may awaken suspicion ; but brain abscesses have sometimes discharged through the tympanum, or through bony fistulæ in the squamous portion of the temporal bone. From the practical point of view it may be said that if a patient has an extradural abscess we may expect him to present more or less strongly pronounced indications for opening up the mastoid process ; or, stated in the reverse way, in any case in which there are present indications of mastoid suppuration, especially if acute otitis media is the primary cause, one is not unlikely to find pachymeningitis externa or extradural abscess, and one should, at the operation, be careful to follow up any necrotic or fistulous tract which may pass to the extra-dural region.

This consideration brings us to inquire, What are the indications or signs which suggest that the mastoid process should be explored in acute suppurative otitis ? In the first place let it be understood that by acute otitis is meant not merely an otitis which has existed a few days. A patient has acute otitis so long as the tympanic cavity and membrana tympani are acutely inflamed, and these parts may continue in a state of acute inflammation for weeks or even months. The state of acute inflammation is obvious on examining the ear with adequate illumination through an aural speculum, and comprises swelling, redness and opacity of the membrana tympani, and of the adjacent parts of the meatus, with concealment of the malleus, and probably with the presence of a pulsating light reflex, with or without a visible perforation of the drum-head.

The indications for opening up the mastoid and antrum are, of course, clear if a typical mastoid abscess is present. The auricle is then pushed outwards and downwards, the fold or crease between the pinna and mastoid is obliterated, and the skin is tense, swollen and fluctuant.

In the absence of the above typical signs of abscess the indications for operation are not so clear. We must all have seen cases of acute otitis, especially in children, in which there was not only pain and tenderness over the mastoid, but even swelling there, due no doubt to periostitis, and yet in such cases recovery has taken place without operation. We must conclude, therefore, that mastoid periostitis may exist without abscess in the mastoid, and may subside under antiphlogistic treatment if there is good drainage from the tympanum through either a spontaneous perforation or an opening made by paracentesis. If a perforation is present and inadequate it should be enlarged by paracentesis.

But if external mastoid periostitis may subside without opening up the bone there is another form of periostitis which indicates clearly that there is suppuration of the antrum, and that operation is required. I refer to periostitis of the deeper portion of the auditory meatus. The swelling from this narrows the meatus in its deeper part, and the swollen part, which has this special significance, is that on the upper and back part of the deep osseous meatus. This swelling is tne so-called "sagging" of the posterior superior meatal wall, that is, swelling of that part of the meatus which lies nearest the antrum. The swelling is really the pointing of an antral abscess, and in some cases an opening forms there, pus escapes, and a fistulous tract is left.

In other cases of acute otitis the indication for operation on the mastoid is the presence of persistent pain, vertigo, tinnitus, vomiting, or nystagmus. The persistence of any one of these symptoms is an indication for operation.

It must be admitted, however, in spite of the help given by such indications as those enumerated, that it is often difficult to decide whether the mastoid should be opened up or not in acute otitis. The operation required in such cases is not a difficult one which demands exceptional skill, but obviously the patient should be spared operation if that is possible with safety. The trouble is that a sudden development of cerebral symptoms may arise in a case of otitis which has been running an almost painless course. The important fact remains to be stated that you may wait with more safety for clear indications of mastoid suppuration before operating in young people than in people of middle or advanced age. In middle life the mastoid process is more likely to be sclerosed. If a mastoid with a thick cortex or a sclerosed cortex suppurates, the suppuration is more dangerous because the pus cannot so easily make its way to the surface and cause periostitis and mastoid abscess. Failing escape in that direction, the pus is likely, unless a timely operation is performed, to spread inwards through the labyrinth or by other routes, and give rise to dangerous intra-cranial troubles.

With respect then to elderly people, the wisest practice is to open up the mastoid if acute suppurative otitis has continued for five or six weeks without distinct signs of abatement, even though there is no mastoid tenderness, and no elevation of temperature. Lastly, in influenza and in diabetes it has to be remembered that dangerous mastoid complications may develop insidiously.

In children and young people the deciding factors for or against operation must be the tendency of the general and local symptoms to increase or decrease from day to day. The timely opening up of the mastoid process is to be regarded as the most important method of preventive treatment of the intra-cranial complications of ear-disease.

(2) **Infective Lateral Sinus Thrombosis.**—This complication is more commonly found in connection with chronic than with acute disease in the temporal bone, and in the great majority of cases the temporal bone itself is affected, not merely the muco-periosteum of the tympanum and mastoid cells. Very frequently there is a perisinus abscess, and this is the cause of the thrombosis. The presence of pus or granulation tissue on the outer wall of the sinus naturally favours thrombosis. Another mode of origin appears to be the spread of thrombi into the sinus from small blood-vessels which pass into it from the temporal bone.

Thrombosis having begun in the lateral sinus, usually at the knee or in the sigmoid portion, tends to extend in various directions. It may spread backwards against the blood-stream towards the torcular, rarely to the longitudinal sinus, and to the transverse sinus of the opposite side. Extension, again, may occur along the superior or inferior petrosal sinuses, and through these into the cavernous sinus of the same side, then possibly to the ophthalmic vein, and to the cavernous sinus of the opposite side. Thirdly, the extension may be with the blood-stream into branches of the sinus, the mastoid emissary vein or the anterior condylar vein, but especially into the jugular bulb and jugular vein, even into the innominate vein, and also into the facial branch of the jugular vein.

Course and Symptoms.—Cerebral symptoms may arise from the presence and irritation of an inflammatory focus within the skull, through circulatory obstruction in the sinus, and possibly through accompanying meningitis serosa. The cerebro-spinal fluid is generally increased in amount. Headache is usual, diffused or limited to the affected side ; vomiting in the early stages is also usual. In severe septicæmic cases delirium is a symptom, but in the cases running a more pyæmic than septicæmic course consciousness is not altered.

The cerebral symptoms are, as in all aural intra-cranial complications, more marked in children than in adults. Optic neuritis is not common in pure sigmoid sinus thrombosis.

The general course of the affection in the organism can be chiefly septicæmic in character or chiefly pyæmic, but it is rarely a pure form of one or the other.

The most characteristic sign of the pyæmic form is the occurrence of rigors with a sudden rise of temperature, followed by a sudden fall. The rigor may last from a quarter to half an hour, or even one to two hours. Sometimes these rigors occur once daily, or there may be two or three days' interval between them, or there may be, on the other hand, two or three rigors in twenty-four hours. A case has been reported, and successfully dealt with, in which no fewer than sixty-five rigors occurred.

The spleen is usually enlarged, the pulse rises and falls with the temperature. Metastatic complications occur most commonly in the lungs only, much more uncommonly in other parts as well as in the lungs, and very rarely in other parts and not in lungs. The sterno-clavicular and shoulder joints are favourite seats of the secondary abscesses, but such may occur in the subcutaneous tissue, in other joints, in bursæ, and in muscles. Sometimes these metastatic inflammations do not end in suppuration. More rarely metastases have been found in the kidneys, spleen and liver.

Here may well be mentioned a clinical type of case to which Körner has directed attention, in which there is otitic pyæmia without sinus phlebitis. The patients are usually children or young people, the ear affection is acute, pyæmic deposits occur in the joints, bursæ and muscles, with absence of such, as a rule, in the lungs. The prognosis is favourable without operation on the sinus.

In the septicæmic type of sinus thrombosis, as distinguished from the pyæmic, the symptoms are those of an acute, rapidly fatal septic poisoning or toxæmia. There is high continuous fever, delirium, and a rapid small pulse ; metastatic abscesses are not characteristic, but septic endocarditis, muscular hemorrhages, retinitis and retinal hemorrhages, nephritis and hepatitis are often present. The spleen is generally enlarged. Death is usual in a few days.

What is the duration and prognosis in cases of sinus thrombosis which have not been operated upon ? Frequently the patient lives two to four weeks—in rare cases longer, even to several months. Death results from such effects as general septicæmia, from pyæmic metastases, especially pyo-pneumothorax, and from meningitis or brain abscess. The patients may recover, and the sinus be permanently obliterated without operation.

Now we come to the all-important questions of diagnosis and treatment. The diagnosis of the well-marked cases presents no difficulty. There is characteristic fever, splenic tumour, and signs of jugular thrombosis. There is a sign of thrombosis known as Griesinger's. It is the presence of localised swelling and œdema at the posterior border of the mastoid, in the position of the mastoid emissary vein. As a matter of fact, this sign is more an indication of an extra-dural abscess than of sinus phlebitis. If this swelling is not limited to the small area mentioned, but also lies over the mastoid process, then it points to mastoid disease only, not to either of the intra-cranial complications mentioned. Irritative or paralytic effects upon the nerves which pass through the condylar foramen or the jugular foramen may give help. The nerves which may be affected are the hypoglossal, spinal accessory, glossopharyngeal and vagus. Their involvement points to an extension of the sinus phlebitis to the foramina mentioned. The septicæmic type of case of sinus phlebitis may resemble typhoid fever, and the pyæmic type may resemble malaria. Examination of the blood will, of course, assist in the differentiation of these maladies.

The diagnosis of less advanced and less typical cases may be difficult. There may, for example, be no local signs or symptoms of a blocked sinus or vein, and the fever may not show the characteristic pyæmic curve. In this connection let it be said that one well-marked rigor should raise a strong suspicion of pyæmia, and that the occurrence of a second rigor should be considered a decisive indication for exploration. In children with meningitic symptoms lumbar puncture will serve to exclude meningitis.

With respect to adults, the rule should be to open up the mastoid and explore the lateral sinus if there is septic ear-disease present, or a recent history of such, and if fever is present of the continuous type not accounted for by the presence of some other disease, *e.g.* typhoid fever, pneumonia, influenza, bronchitis or tonsillitis, etc. The septicæmic form of sinus thrombosis will very probably be found to exist in such circumstances. Further, in such cases, as well as in cases with pyæmic fever, an absence of mastoid tenderness and swelling must never be considered to contra-indicate operation. I feel I cannot lay too much stress on this point. I am convinced that I have myself delayed operation to the detriment of patients more than once, because there was no tenderness or swelling of the mastoid process, and I may be tempted to do so again. I have also seen house-surgeons and practitioners repeatedly fail to recognise the need for operation because there has been neither pain, heat, swelling nor tenderness over the mastoid process. No trouble or care must, of course, be spared to exclude other possible sources of the fever before operating.

The difficulty of diagnosis is increased by the fact that mastoiditis may be present when the otitis which caused it is subsiding or has subsided. In still rarer cases there has not been a perforation of the drum, and no inflammation of that structure has been previously recognised, nor is recognisable at the time of examination.

Lastly, let me say exploration is justified and desirable when there is a fair probability of the presence of septic sinus thrombosis, and that a careful exploration of the sinus is not a dangerous procedure.

To sum up. If in the course of a case of acute purulent otitis media (not at the beginning), or in a case of chronic purulent otitis, there is a sudden rise of temperature, say to 102° or more, accompanied by a rigor, with headache and malaise, and if non-aural causes for the temperature can be excluded, and there are no symptoms of meningitis or cerebral abscess, the mastoid should be opened up and the sinus exposed without delay. Children are an exception to the rule, for they may have a high temperature for a fortnight from acute otitis without complications.

If under such circumstances the exposed sinus is found tense and elastic one should wait to see the effect of the mastoid operation. Most likely it will prove sufficient, and the patient will recover without thrombosis. If the temperature remains high during the following days, or if a rigor occurs, then the interior of the sinus must be explored.

We must now consider the treatment of the sinus when exposed in further detail. Some authorities state that you may have a depressed appearance of the sinus from compression, and a benign thrombus in it which is best left undisturbed. I should myself be too doubtful about the non-infective state of the thrombus to leave it undisturbed in such circumstances. The exposure of the sinus in the search for possible thrombosis should be free. The thrombosed part may be low down in the sigmoid sinus, between the lower bend and the jugular bulb, or the sinus itself may be free from thrombus, and yet the jugular bulb contain septic clot, infected perhaps through the floor of the tympanic cavity. I may state at once that I have never myself exposed the jugular bulb either to examine it for thrombus or to remove thrombus known to be there. I content myself with exposing the sigmoid sinus low down, and passing a curette from the lowest point exposed towards the jugular bulb from above. The spoon will not enter the bulb, but will pass to a point near it. To clear the bulb itself from clot I have hitherto relied upon passing a curette upwards into it from the jugular vein below. I consider syringing through from vein to sinus rather risky, for the dura might give way, and meningitis result. Operations for exposure of the jugular bulb have been specially described by Grunert and Piffl and Voss. A great drawback to those operations is the proximity of the facial nerve.

It is not always easy to decide whether there is thrombosis of the exposed sinus or not. If present the thrombus may block the lumen of the sinus, or be merely a parietal layer not blocking the lumen. The use of a needle and syringe for exploration for clot is safer than incision, and may well be employed first. If it does not yield sufficient information the sinus must be opened. If the needle shows the sinus to be blocked it must be freely laid open. The clot may be obviously septic in the middle part, but apparently ending above and below in healthy clot. Some surgeons leave healthy-looking clot undisturbed, and remove only the broken-down portions. This appears to me to be taking too much risk of a continuation of septic thrombosis. You cannot be sure of the asepticity of the clot left behind.

If the exploratory needle withdraws healthy blood, normal in appearance and free from odour, we are faced with several possibilities. There may be parietal clot, not occluding the lumen. In my opinion the mere suspicion of that being the state of affairs does not justify opening the sinus and tying the jugular vein. I believe parietal clotting not infrequently occurs in cases which recover without opening up the sinus. Then the exploring syringe may draw off blood because the clotting is in the jugular bulb only. There are tests for the presence or absence of this condition—*e.g.* Whiting's test—but they fail to prove the presence or absence of a parietal clot in the bulb. There is only one way of proving the presence or absence of septic clot in the bulb—that is, by exposing and opening it. If in a particular case I felt fairly sure from the symptoms and signs that the bulb was infected, I should treat the case as one with thrombosis of the jugular vein, *i.e.* I should open the sigmoid sinus and jugular vein, and clear out both with the curette as well as possible, and drain both, and I have, of course, repeatedly done this. But some advocate more radical procedures; for example, laying open through one curved continuous incision the sinus, jugular bulb, and jugular vein.

The last matter in connection with the treatment of sinus thrombosis about which I wish to speak is the question of ligaturing the jugular vein. Some surgeons always tie the vein if thrombosis exists, others only in certain cases.

The following facts bear upon the question :—

(1) Almost as many cases of sinus thrombosis have been successfully treated without the ligature as with it. Statistics perhaps show a slight advantage from applying the ligature.

(2) Though ligature of the vein closes the main channel for the transmission of septic emboli to the lungs and other parts, it does not close all channels. After ligation fresh infective material may, and does sometimes, pass to the heart from emissary veins, from the petrosal sinuses, or from the torcular end of the lateral sinus.

(3) The operation of ligature of the jugular takes time, and makes another wound, usually septic, and leaves a scar.

(4) The operation has certain dangers, viz. (1) it has been quickly followed sometimes by extension of thrombosis into the inferior petrosal sinus, and thence into the cavernous sinus; (2) the sudden stoppage of the flow of blood through the vein may lead to congestive œdema and necrosis of the brain. This is only likely if the other lateral sinus is abnormally narrow. Further, this danger cannot result from ligation if the vein or sinus is already blocked with clot. But many writers have advocated tying the vein for non-obliterating thrombosis.

In my own opinion the patient's best interests will be served if this main channel of embolic invasion is closed in nearly all cases. I would make an exception if the extent of the thrombosis in the sigmoid sinus was small, and healthy sinus wall could be seen above and below the diseased portion, and free bleeding could be established from both the torcular and jugular end of the infected sigmoid by curetting. I would not trust any clot in the sinus which obstructed the free flow of blood from both directions. If ligatured at all, the best time to place the ligature is immediately after the diagnosis is established, whether by palpation in the neck, or more usually by inspection of the sinus through the opening in the skull. It seems to me best to resect a portion of the vein to a level above the facial branch, after dividing that branch, but to leave the upper end of the jugular vein long enough to be brought to the surface in the upper angle of the skin wound for drainage purposes.

### DISCUSSION.

Dr. Carey Coombs, after congratulating the Society on the excellence of the two introductory papers, said that a brief review of the autopsy figures of the General Hospital would form the basis of his remarks. There were thirty-four cases in which intracranial complications of middle ear inflammation had proved fatal. In these cases sinus thrombosis was found seventeen times, abscess in the substance of the brain, or cerebellum, in fifteen cases (temporo-sphenoidal lobe nine, cerebellum five, both temporal lobe and cerebellum one). In three cases there was a surface abscess, extra or sub-dural, and in only sixteen cases was diffuse meningitis noted. This latter was most often basic, and was usually accompanied by one of the more localised lesions mentioned above ; presumably in some cases meningitis had only occurred subsequently to abscess formation or sinus thrombosis. He had been surprised to find the incidence of meningitis so small. Testing the matter from another point of view, he had found that only five out of fifty cases of fatal meningitis were otitic in origin. The main inference drawn from these figures was that in most cases there was a stage during which the infective process within the skull remained localised in its effects, and therefore open to a successful surgical attack. With regard to successful treatment of meningitis (if it were indeed possible, as has been claimed of late), early diagnosis would always be the important factor. He mentioned rigidity of the neck with tenderness of the sub-occipital muscles and polymorphonuclear leucocytosis in the cerebro-spinal fluid as valuable signs by reason of their early appearance. He asked two questions : What was the evidence on which it was claimed that such a phenomena as " serous meningitis " occurred ? and Was it possible, in the experience of members of the Society, to save lives threatened by a spreading meningitis by early operation ?—Mr. Hey Groves pointed out that in view of the uncertain diagnosis in many cases operative exploration of the lateral sinus and cerebrum and cerebellum, on the lines advocated by Mr. Percy Dean, was often advisable.—Dr. Stack related a case in which the symptoms resembled in every way cerebral abscess following mastoid disease. On four different occasions the brain was explored, altogether in about twenty directions, including both cerebella, both temporo-sphenoidal and both frontal lobes. No abscess was found. The case was presumed, therefore, to be œdema. Dr. J. O. SYMES referred to the rarity of intra-cranial lesions with acute otitis, although frequently there were transient attacks of cerebritis or meningitis clearing up without operation. Probably all cases showing optic neuritis should have a mastoid operation performed, and if improvement did not result, then later the dura should be incised. He compared the symptoms of meningitis and those of cerebral abscess, the distinctive points in the latter being the regular, slow, full pulse, low temperature (in a cerebral abscess there is a rise of temperature on the opposite side of the body), motor paralysis, perhaps with loss of localisation of touch, slow disappearance of the superficial reflexes on one side only, and unilateral optic neuritis with moderate swelling of the disc. He described a case of cavernous sinus thrombosis, which made a good recovery, and mentioned that when vaccine therapy had to be employed in intra-cranial suppuration the vaccine was generally a compound one. In a case of cerebellar abscess under his care the organisms were a bacillus, a diplococcus, and the bacillus pyocyaneus; in a case of meningitis the organisms were a staphylococcus and the pneumococcus. — Dr. WATSON-WILLIAMS remarked on the difficulties that many cases of intra-cranial infection secondary to aural suppuration presented—firstly in determining the existence of intra-cranial lesions, and secondly in differentiating between sinus or perisinus suppuration, sub-dural or lepto-meningeal abscess, and abscesses of the cerebrum or cerebellum; and illustrated these points by citing various cases in which these difficulties arose, and on which he had been called to operate from time to time. One group to which he directed notice consisted of those cases where acute complications arose in chronic purulent otitis in both ears. One had then to determine which ear was the source of the intra-cranial complication, the diffculties in differentiation being greatly increased when the patient was semi-conscious. He believed that one of the most valuable differential diagnostic aids was the presence of optic neuritis restricted to or most marked in one disc, and the ipso-laterality of the intra-cranial lesion was sometimes the only guide as to the side requiring intra-cranial exploration.—Mr. A. J. Wright pointed out that it had been estimated that 7 per cent. of the cases of suppurative ear disease had optic neuritis.—Mr. C. H. Walker and Dr. Walker Hall joined in the discussion.

REPLY.

In reply, Dr. Michell Clarke said that Dr. Coombs's statistics with a fewer number of cases fairly corresponded in proportionate frequency of affections considered to those given by Dr. Logan Turner at the discussion in London. He had not had enough personal experience to allow him to express an opinion as to serous meningitis. In the cases of diffuse septic meningitis he had seen the issue was lethal. Dr. Stack's case was a good illustration of the difficulties of diagnosis in intra-cranial abscess. With regard to optic neuritis, he thought that if an individual case it was of unequal severity on the two sides that point was of more importance in the differential diagnosis than the intensity of the swelling. In reply to Dr. Edgeworth, the plantar reflex in an uncomplicated lesion of a lateral lobe of the cerebellum would be of the flexor type. Dr. Wright's statement that optic neuritis was present in seven per cent. of cases of middle ear suppuration was one of the points which he had hoped would be brought out in the discussion, and was of great importance. There was no doubt that the public were still much too careless about chronic otorrhœa.

